# Bone marrow transplantation induces changes in the gut microbiota that chronically increase the cytokine response pattern of splenocytes

**DOI:** 10.1038/s41598-022-10637-7

**Published:** 2022-04-27

**Authors:** Saeed Katiraei, Janna A. van Diepen, Luciana P. Tavares, Lisa R. Hoving, Amanda Pronk, Ineke Verschueren, Patrick C. N. Rensen, Jaap Jan Zwaginga, Sarantos Kostidis, Martin Giera, Mauro Teixera, Ko Willems van Dijk, Mihai G. Netea, Jimmy F. P. Berbée, Vanessa van Harmelen

**Affiliations:** 1 Department of Human Genetics, Leiden University Medical Center, Einthovenweg 20, 2333 ZC Leiden, The Netherlands; 2 Einthoven Laboratory for Experimental Vascular Medicine, Leiden University Medical Center, Leiden, The Netherlands; 3 Department of Internal Medicine, Radboud UMC, Nijmegen, The Netherlands; 4 Laboratory of Immunopharmacology, Department of Biochemistry and Immunology, Universidade Federal de Minas Gerais, Belo Horizonte, Brazil; 5 Division of Endocrinology, Department of Medicine, Leiden University Medical Center, Leiden, The Netherlands; 6 Department of Immunohematology and Blood Transfusion, Leiden University Medical Center, Leiden, The Netherlands; 7 Center for Proteomics and Metabolomics, Leiden University Medical Center, Leiden, The Netherlands

**Keywords:** Antimicrobial responses, Bone marrow transplantation, Interleukins, Bacterial host response

## Abstract

Bone marrow transplantation (BMT) involves conditioning regimens which acutely induce side effects, including systemic inflammation, intestinal damage and shifts in the gut microbial composition, some of which may persist chronically. As the gut microbiota affect systemic immune responses, we aimed to investigate whether, post-BMT, the peripheral immune system is modulated as a direct consequence of alterations in the gut microbiota. We show that 24 weeks post-BMT, splenocytes but not peritoneal macrophages display increased cytokine response patterns upon *ex-vivo* stimulation with various pathogens as compared to untreated controls. The pattern of BMT-induced cytokine responses was transferred to splenocytes, and not to peritoneal macrophages, of healthy controls via co-housing and transferred to germfree mice via transplantation of cecum content. Thus, BMT induces changes in gut microbiota that in their turn increase cytokine responsiveness of splenocytes. Thus, BMT establishes a dominant microbiota that attenuates normalization of the immune-response.

## Introduction

The intestine is not only the site where food digestion takes place, but it is also home to more than 10^14^ commensal microorganisms that are collectively called the gut microbiota. The microbiota continuously interact with and provide benefits to the host, as they are involved in processes such as fermentation of indigestible fibres, metabolism of xenobiotics and regulation of the immune system. Studies in germfree mice that lack gut microbiota have provided insight into the role of gut microbiota in regulating the immune system. For example, germfree mice have a dysfunctional mucosal immune system with less and smaller Peyer’s patches and mesenteric lymph nodes^1,2^. These mice also have reduced numbers of intra-epithelial lymphocytes with a compromised immune function^3^. The gut microbiota are thus important for shaping a functional mucosal immune system.

In addition to affecting the mucosal immune system, recent studies have demonstrated that the gut microbiota are important in the development and function of the immune system beyond the intestine. For example, the gut microbiota play a role in the pathogenesis of autoimmune disorders such as rheumatoid arthritis and type-1 diabetes^2,4,5^. Clarke et al*.*^6^ reported that bacterial components translocate from the gut into the circulation under basal conditions and serve as mediators that systemically prime neutrophils in the bone marrow. However, the effects of the gut microbiota on other key lymphoid tissues such as the spleen have not been fully characterized. Germfree mice have reduced numbers of CD4+ cells and smaller germinal centers within the spleen as well as lower systemic antibody levels^4,7^. Moreover, oral administration of dietary fibres or short-chain fatty acids (SCFA) to mice increases antibody responses by B cells in the spleen^8^, which indicates that metabolites from the gut microbiota affect spleen function.

Bone marrow transplantation (BMT) is applied as therapy for patients with specific cancers of the bone marrow or blood, such as multiple myeloma or leukemia. To minimize residual disease, to create space for the transplant, and to achieve immune-ablation, BMT is preceded by conditioning regimens like total body irradiation (TBI) or chemotherapeutic agents. A drawback of these conditioning regimens is that they induce acute and chronic side effects. TBI not only ablates bone marrow cells, but may also cause acute damage to the gastrointestinal tract. This damage promotes the leakage of bacterial components from the gut into the systemic circulation. Bacterial components act as toll-like receptor ligands, activate immune cells and thus cause systemic inflammation^9^. This phenomenon may certainly explain some of the acute side effects of the BMT treatment.

However, BMT treatment not only resets the host immune system but also affects host microbiota^10^. The long term composition and activity of the intestinal microbiota is the result of intricate interactions between bacteria, host and environment. It is more than likely that BMT treatment thus results in permanently altered microbiota that may contribute to changes in the intra- and extra-intestinal immune system of the host^11^.

In the current study, we investigated in mice the effect of BMT on the response patterns of splenocytes and peritoneal macrophages to various pathogenic stimuli as markers of the extra-intestinal immune system. Splenocytes are a mixed population of immune cells and thus represent responses from both the adaptive and innate immune system, whereas peritoneal macrophages represent the innate immune system. The potential role of microbiota in the response patterns of splenocytes and peritoneal macrophages was investigated by co-housing control mice with BMT-treated mice and by transfer of cecum content to germfree mice. Our data show that the BMT-induced increase in the cytokine response pattern of splenocytes to pathogenic stimuli can be transferred via the gut microbiota.

## Results

### The cytokine response pattern of stimulated splenocytes was increased after BMT and transferred to healthy controls via co-housing

To study the effect of BMT on the response patterns of splenocytes and peritoneal macrophages to various pathogenic stimuli and the potential role of microbiota herein, C57BL/6 mice underwent syngeneic BMT or sham procedure and were co-housed in three combinations to arrive at the following four groups: (1) healthy control mice co-housed with healthy control mice, (2) BMT-treated mice co-housed with other BMT-treated mice, (3) BMT-treated mice co-housed with healthy control mice, and (4) healthy controls co-housed with BMT-treated mice. Group 1 served as control for the other three groups. All mice, both BMT-treated and control mice, received water containing antibiotics and were exposed to the same dietary regimen. After recovery from the BMT procedure, mice were fed a low-fat diet for 16 weeks to determine the chronic effects of microbiota exchange via co-housing on the responses of splenocytes and peritoneal macrophages. At the end of the study, peritoneal macrophages were isolated and the spleen was harvested and cells were immediately used for ex vivo stimulation assays.

To assess the general health condition of the mice after BMT, we monitored body weight during the study. As reported previously^12–14^, the BMT procedure reduced body weight already within a few days (Supplementary Fig. S1). The BMT-induced decrease in body weight was not restored by co-housing the mice with healthy controls and vice versa the body weight of the healthy co-housed controls was not affected by co-housing with BMT-treated mice.

**Figure 1:**
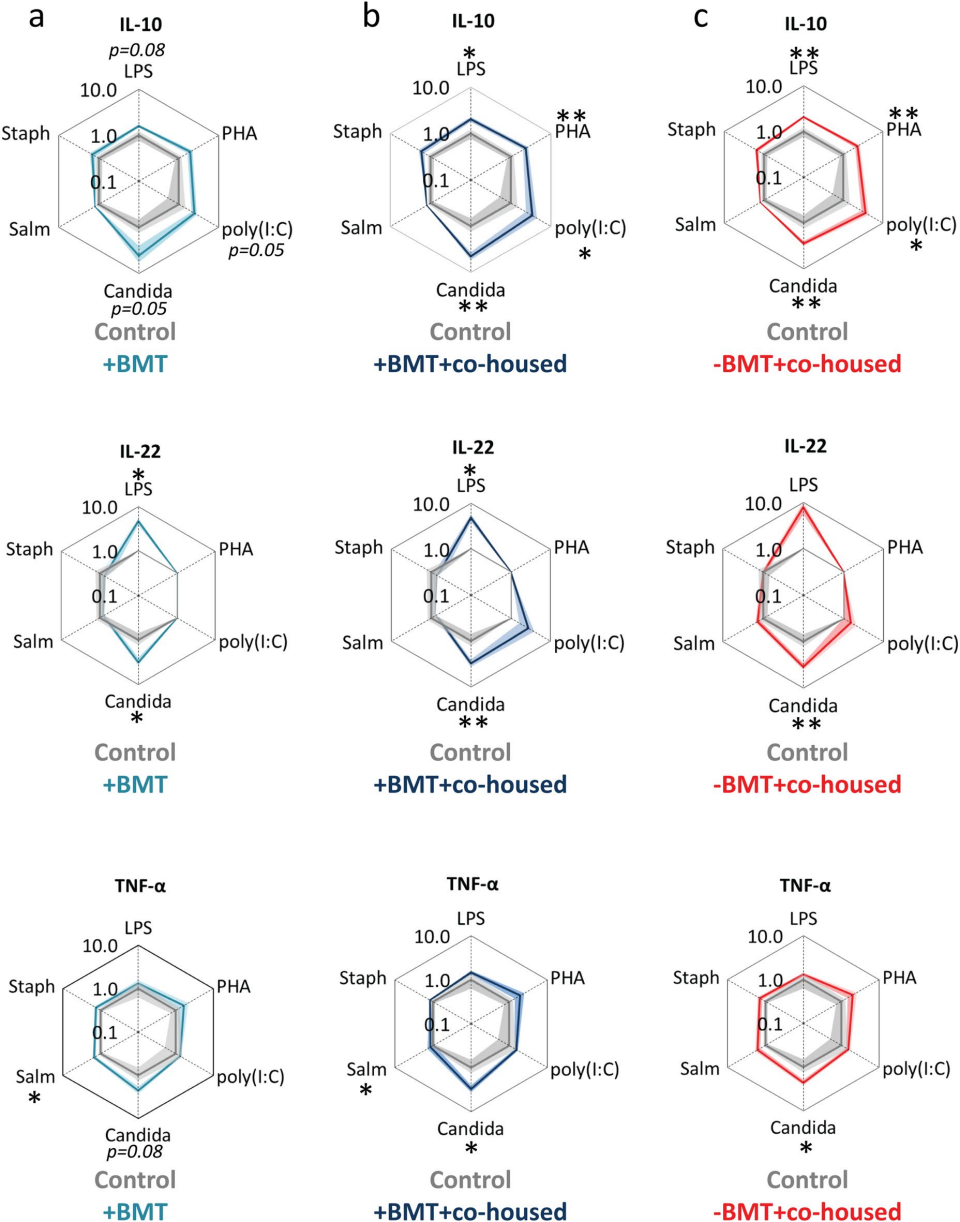
The effect of co-housing after BMT on cytokine secretion of stimulated splenocytes. Spiderplots show IL-10, IL-22 and TNF-α release in response to various pathogenic stimuli from splenocytes derived from (**a**) BMT-treated mice co-housed with BMT mice (outer light blue lines), (**b**) BMT-treated mice co-housed with healthy control mice (outer dark blue lines), and (**c**) control mice co-housed with BMT-treated mice (outer red lines). Cytokine concentrations in response to the stimuli are normalized to the cytokine concentrations of healthy control mice co-housed with control mice (inner grey lines), averaged per group and plotted on a log scale. Every corner of the spiderplot hexagon represents the response to one stimulus. The data lines and shades represent means and SEM, respectively; Groups were compared using Mann–Whitney U test; n = 5–6 per group; *p < 0.05; **p < 0.01. Candida, *Candida conidia*; LPS, lipopolysaccharide; PHA, Polyhydroxyalkanoates; poly(I:C), Polyinosinic:polycytidylic acid; Salm, *Salmonella typhimurium*; Staph, *Staphylococcus aureus*.

Splenocytes of BMT-treated mice showed higher IL-10, IL-22 and TNF-α cytokine release as compared to healthy controls, when stimulated with various pathogenic stimuli such as LPS, Polyinosinic:polycytidylic acid (Poly(I:C)), *Candida conidia* or *Salmonella typhimurium* (outer light blue lines versus inner grey lines, Fig. 1a). Similar IL-10, IL-22 and TNF-α cytokine response patterns were seen in the BMT-treated mice that were co-housed with healthy control mice (outer dark blue lines versus inner grey lines, Fig. 1b), suggesting that co-housing with healthy controls did not rescue the hyper-responsive phenotype of the splenocytes induced by BMT. Strikingly, the healthy controls that were co-housed with the BMT-treated mice also showed a similar IL-10, IL-22 and TNF-α response pattern upon pathogenic stimulation (outer red lines versus inner grey lines Fig. 1c), indicating that the BMT-induced responsiveness of the splenocytes was transferred to healthy controls via co-housing.

### The cytokine response pattern of stimulated peritoneal macrophages was not affected after BMT

**Figure 2:**
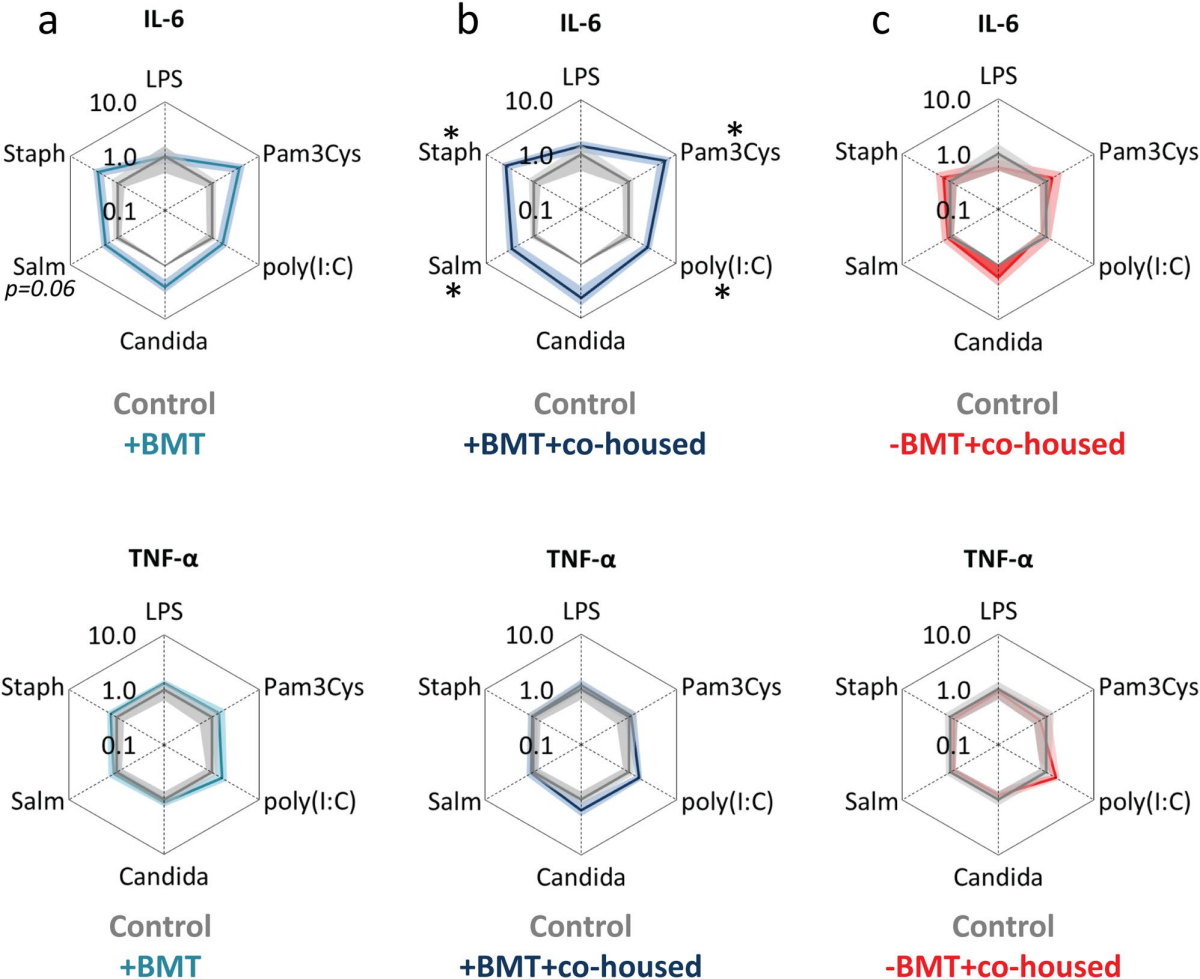
The effect of co-housing after BMT on cytokine secretion of stimulated peritoneal macrophages. Spiderplots show IL-6 and TNF-α release in response to various pathogenic stimuli from peritoneal macrophages derived from (**a**) BMT-treated mice co-housed with BMT mice (outer light blue lines), (**b**) BMT-treated mice co-housed with healthy control mice (outer dark blue lines), and (**c**) control co-housed mice co-housed with BMT-treated mice (outer red lines). Cytokine concentrations in response to the stimuli are normalized to the cytokine concentrations of healthy control mice co-housed with control mice (inner grey lines), averaged per group and plotted on a log scale. See legend to Fig. 1 for more information. Groups were compared using Mann–Whitney U test; n = 6; *p < 0.05.

Peritoneal macrophages of BMT-treated mice did not show increased cytokine release upon ex vivo stimulation with various stimuli, except for a tendency towards increased IL-6 release upon stimulation with *S. typhimurium* stimulation (outer light blue lines versus inner grey lines, Fig. 2a). Peritoneal macrophages of BMT-treated mice that were co-housed with healthy control mice released more IL-6 after stimulation, which was significant for Pam3Cys, poly(I:C), *S. typhimurium* and *Staphylococcus aureus* (outer dark blue lines versus inner grey lines, Fig. 2b). Although the cytokine response pattern of splenocytes upon different pathogenic stimuli was similar between the two groups of BMT-treated mice (Fig. 1a,b), the IL-6 cytokine response pattern of macrophages from co-housed BMT-treated mice was different (Fig. 2b). For TNF-α secretion, no differences were observed between the different groups (Fig. 2a–c). These data indicate that the cytokine response pattern of peritoneal macrophages upon stimulation was not transferred from BMT-treated mice to control mice via co-housing.

### The cytokine response pattern after BMT was transferred to splenocytes of germfree mice after transplantation of cecum content

**Figure 3:**
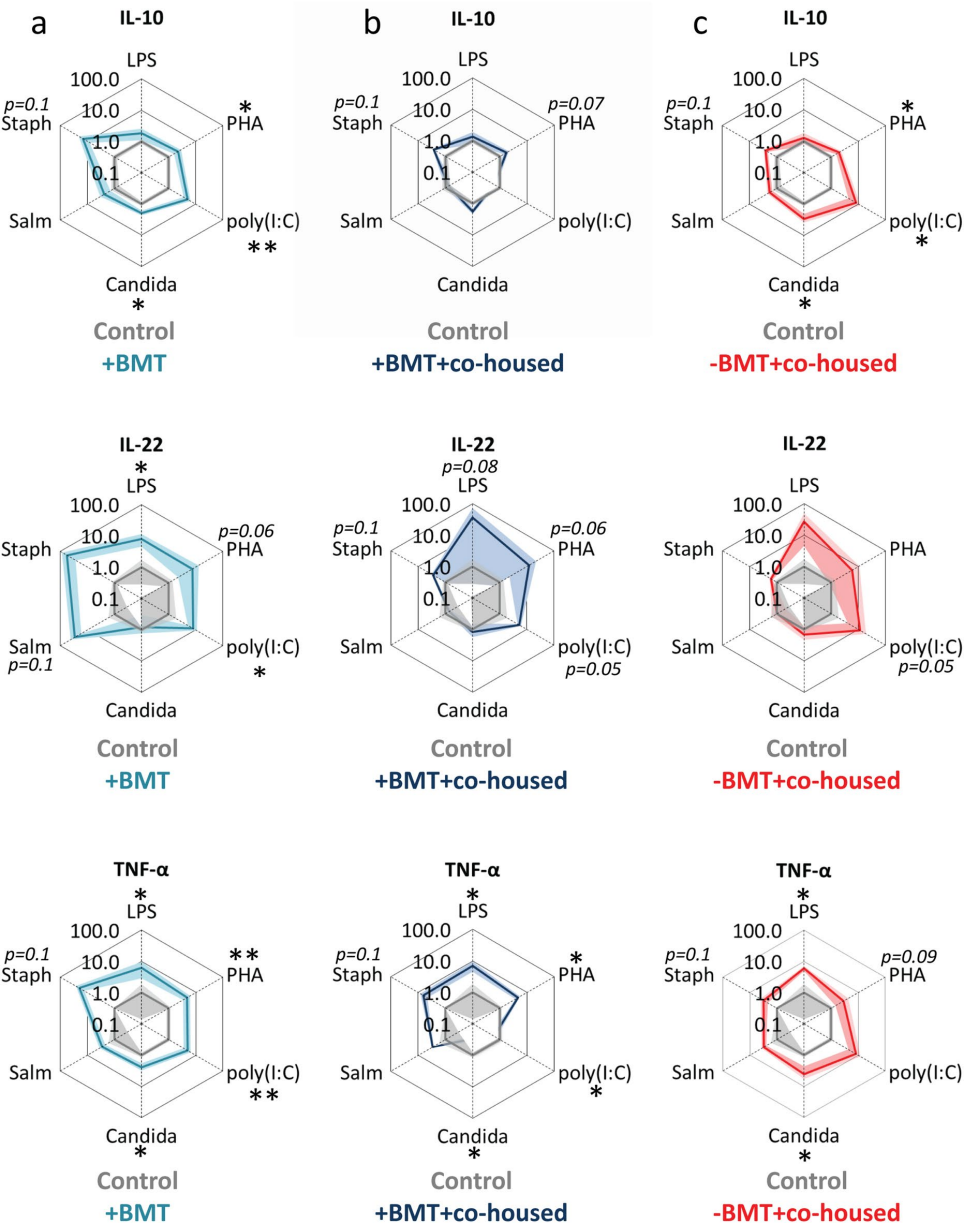
The effect of cecum content transfer after BMT on cytokine secretion of stimulated splenocytes. Spiderplots show IL-10, IL-22 and TNF-α release in response to various pathogenic stimuli from splenocytes derived from (**a**) germfree mice inoculated with cecum content samples of BMT-treated mice co-housed with BMT-mice (outer light blue lines), (**b**) germfree mice inoculated with cecum content samples of BMT-treated mice co-housed with control mice (outer dark blue lines), and (**c**) germfree mice inoculated with cecum content samples of control mice co-housed with BMT mice (outer red lines). Cytokine concentrations in response to stimuli are normalized to the cytokine concentrations of splenocytes from germfree mice inoculated with cecum content samples of control mice co-housed with control mice (inner grey lines). See legend to Fig. 1 for more information. Groups were compared using Mann–Whitney U test; n = 3–5; *p < 0.05.

To determine whether intestinal microbiota were causally involved in the altered cytokine response pattern by splenocytes after BMT, germfree mice were inoculated with the cecum content of BMT-treated mice co-housed with or without control mice or with the cecum content of co-housed control mice. Splenocytes derived from germfree mice that were colonized with cecum content of BMT-treated mice as compared to those colonized with cecum content of control mice, secreted more cytokines after stimulation with various pathogenic stimuli, which reached significance for LPS (IL-22 and TNF-α), PHA (IL-10 and TNF-α), poly(I:C) (IL-10, IL-22 and TNF-α) and *C. conidia* (IL10 and TNF-α) (outer light blue lines versus inner grey lines, Fig. 3a). A partly overlapping cytokine response pattern was observed in germfree mice inoculated with cecum content of BMT-treated mice co-housed with BMT mice and in germfree mice inoculated with cecum content of BMT-treated mice co-housed with control mice (outer light and dark blue lines versus inner grey lines, Fig. 3a,b), with similar (trends in) responses of IL-10, IL-22 and TNF-α secretion to PHA stimulation. Remarkably, splenocytes derived from germfree mice colonized with cecum content of control mice co-housed with BMT-treated mice showed a cytokine response pattern very similar to splenocytes from germfree mice that were colonized with cecum content of BMT-treated mice (outer red lines and light blue lines versus inner grey lines, Fig. 3a,c). The IL-10 response to PHA, poly (I:C) and *C. conidia* were identical, as was the TNF-α response to LPS and *C. conidia.* These data are in line with the results derived from the co-housing experiment and indicate that the increased responsiveness of splenocytes after BMT can be largely transferred by cecum content transplantation.

**Figure 4:**
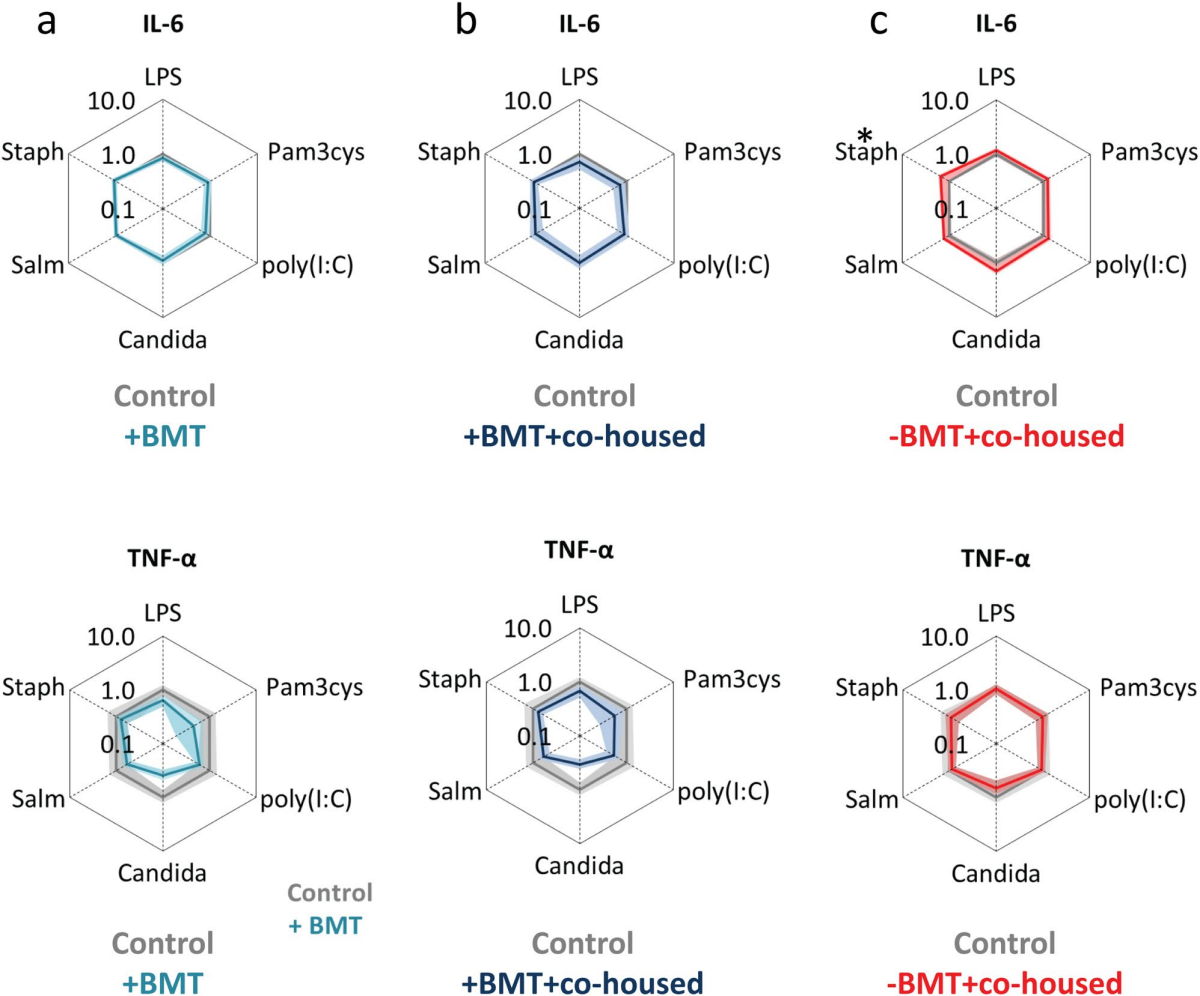
The effect of cecum content transfer after BMT on cytokine secretion of stimulated peritoneal macrophages. Spiderplots show IL-6 and TNF-α release in response to various pathogenic stimuli from peritoneal macrophages derived from (**a**) germfree mice inoculated with cecum content samples of BMT-treated mice co-housed with BMT-mice (outer light blue lines), (**b**) germfree mice inoculated with cecum content samples of BMT-treated mice co-housed with control mice (outer dark blue lines), and (**c**) germfree mice inoculated with cecum content samples of control mice co-housed with BMT mice (outer red lines). Cytokine concentrations in response to stimuli are normalized to the cytokine concentrations of peritoneal macrophages from germfree mice inoculated with cecum content samples of control mice co-housed with control mice (inner grey lines). See legend to Fig. 1 for more information. Groups were compared using Mann–Whitney U test; n = 4–5.

Peritoneal macrophages of germfree mice inoculated with cecum content of BMT-treated mice and co-housed BMT-treated mice did not show increased IL-6 and TNF-α secretion upon stimulation (outer light blue and dark blue lines versus inner grey lines, Fig. 4a–c). Peritoneal macrophages of germfree mice inoculated with cecum content of co-housed controls only showed a minor increase in IL-6 secretion upon *S. aureus* incubation (outer red lines versus inner grey lines, Fig. 4c). These data indicate that gut microbiota specifically stimulate cytokine secretion of splenocytes but not of peritoneal macrophages after BMT.

### Splenocytes of germfree mice showed lower cytokine secretion compared to conventional mice

**Figure 5:**
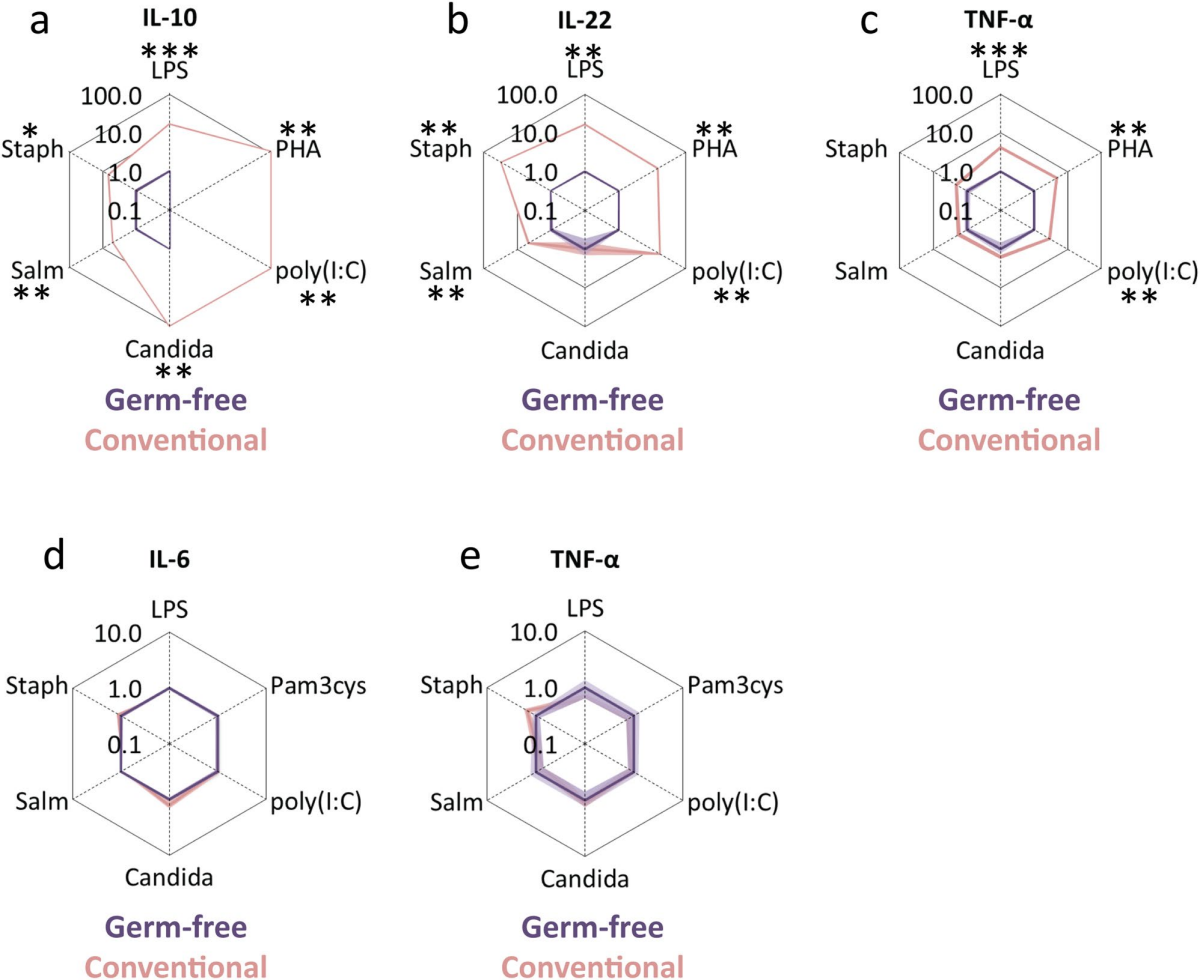
Cytokine secretion of stimulated splenocytes and peritoneal macrophages from germfree versus conventionally housed mice. Spiderplots show (**a**) IL-10, (**b**) IL-22 and (**c**) TNF-α release from splenocytes of germfree mice (inner purple lines) upon stimulation with different stimuli compared to splenocytes of conventional mice (outer pink lines). Spiderplots show (**d**) IL-6 and (**e**) TNF-α release of peritoneal macrophages of germfree mice (inner purple lines) upon stimulation, compared to conventional mice (outer pink lines). Cytokine concentrations in response to the stimuli are normalized to the cytokine concentrations of splenocytes of germfree mice (inner grey lines), averaged per group and plotted on a log scale. See legend to Fig. 1 for more information. Values are means ± SEM; Groups were compared using Mann–Whitney U test; n = 4–9; *p < 0.05; **p < 0.01; ***p < 0.001.

To further investigate the role of gut microbiota in the responsiveness of splenocytes and macrophages, we compared cytokine response pattern of ex-vivo stimulated splenocytes and peritoneal macrophages derived from conventionally versus germfree raised mice. Splenocytes from conventional mice secreted more IL-10, IL-22 and TNF-α as compared to untreated germfree mice upon stimulation with LPS, Polyhydroxyalkanoates (PHA) and poly(I:C), and more IL-10 and IL-22 upon stimulation with *S. typhimurium* and *S. aureus* (outer pink lines versus inner purple lines, Fig. 5a–c). These data indicate that the presence of gut microbiota increases the cytokine response pattern of splenocytes upon pathogenic stimulation. In contrast to splenocytes, there was no increase of cytokine secretion by ex-vivo stimulated peritoneal macrophages between conventional mice and untreated germfree mice (outer pink lines versus inner purple lines, Fig. 5d,e). These data confirm that cytokine secretion of stimulated peritoneal macrophages is not affected by the presence of gut microbiota.

### 16S sequencing did not show consistent longitudinal effects of BMT on intestinal microbiota composition

**Figure 6:**
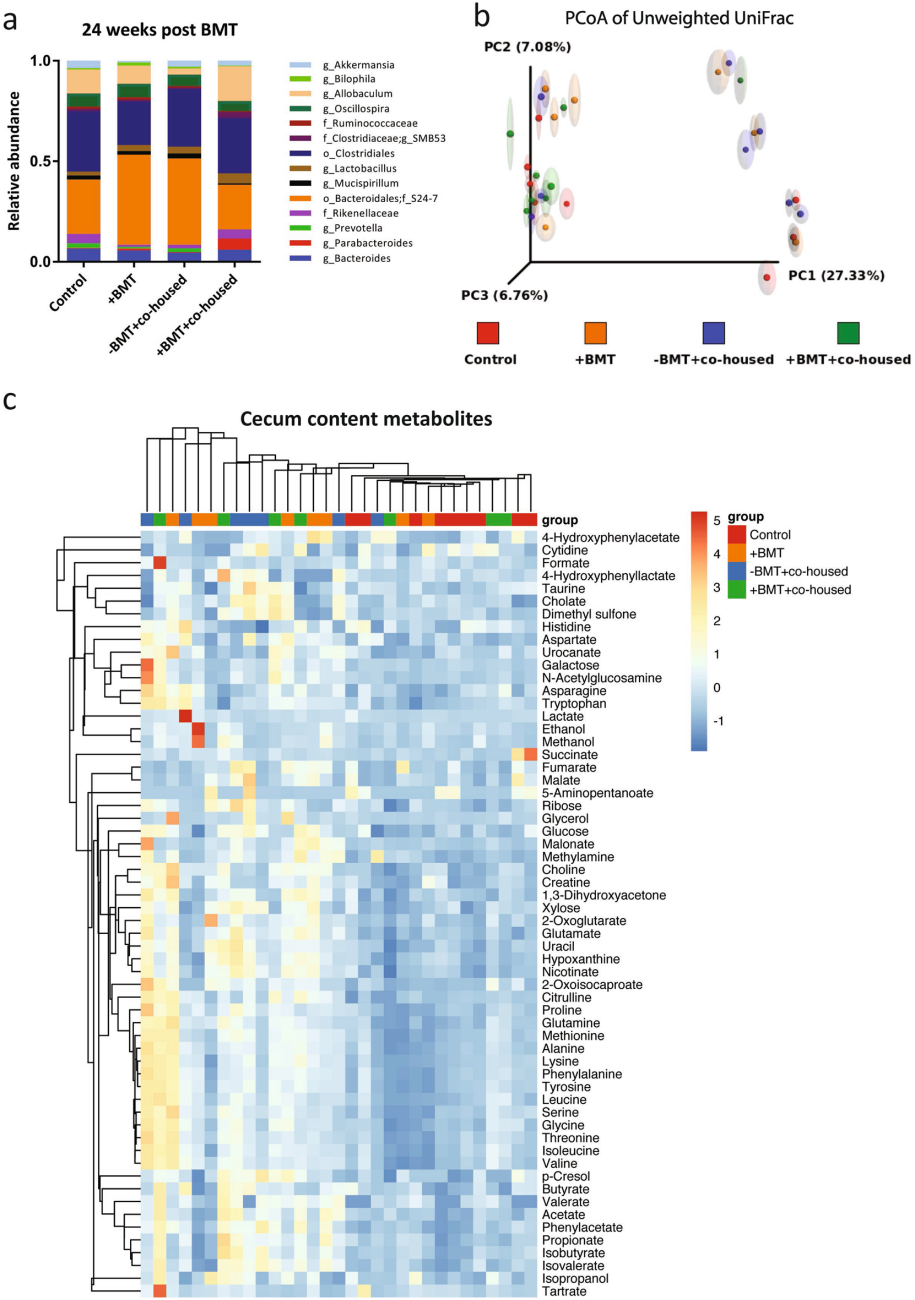
The effect of BMT on fecal microbiota composition and metabolites in the cecum. (**a**) Relative abundance of fecal microbiota in each experimental group as determined by 16S rRNA gene sequencing 24 weeks after BMT. (**b**) Unweighted UniFrac-based principal coordinate analysis (PCoA) of bacterial communities in fecal samples 24 weeks after BMT. Each dot represents one mouse, each colour represents one experimental mouse group; n = 7–8 per group. (**c**) Heatmap of metabolites in cecum content measured by 1H-NMR; n = 7–9 per group.

To investigate potential changes in gut microbiota composition induced by BMT that may explain the increased cytokine response pattern of the splenocytes, we determined the intestinal microbial composition by 16S sequencing. We sequenced fecal samples 24 weeks post-BMT, which was just prior to the ex-vivo splenocyte stimulations and collection of the cecum contents. Bacterial taxa were not significantly different between BMT-treated and control mice, either with or without co-housing (Fig. 6a). There was a large inter-individual variation between the individual samples from the different groups as revealed by the PCoA plots of unweighted UniFrac distance (Fig. 6b). Individual samples from the different treatment groups clustered together in three sub-clusters, but no clustering was observed based on treatment. The splenocyte responsiveness could thus not be linked to obvious BMT-induced differences in intestinal microbial composition as determined by 16S sequencing.

### Co-housing with BMT-treated mice affected cecum metabolite profiles of healthy control mice

To investigate whether BMT induced alterations in gut microbial function that may explain the cytokine response pattern of the splenocytes, we measured 61 metabolites in cecum content samples by 1H-NMR. Metabolite concentrations were quantitatively measured and plotted as z scores in a heat map after unsupervised clustering of all mice (Fig. 6c). The metabolite profile patterns showed considerable heterogeneity between the four experimental groups (Fig. 6c). However, the mice from the control group are largely clustered together and are overall different from the other groups. These data indicate that BMT-treated and healthy control mice modulate each other’s metabolites via co-housing. However, none of the metabolites were clearly linked to the cytokine responses pattern of splenocytes of BMT-treated mice.

## Discussion

Our results demonstrate that BMT induced chronically increased cytokine responses of splenocytes but not peritoneal macrophages upon *ex-vivo* stimulation with various pathogenic stimuli. For splenocytes, and not for peritoneal macrophages, the BMT-induced cytokine response patterns were at least partly mediated via the gut microbiota, as the increased response pattern was largely transferred to splenocytes of healthy control mice via co-housing and via cecum content transplantation to germfree mice. In line with a role for microbiota, splenocytes, but not peritoneal macrophages from germfree mice showed lower stimulated cytokine response patterns as compared to conventional mice. The BMT-induced increased splenocyte response pattern could not be linked to obvious BMT-induced differences in gut microbiota composition or metabolites in cecum. Nevertheless, our data clearly show that gut microbiota are an important determinant of the cytokine response pattern of splenocytes, but not of peritoneal macrophages.

The differential role of microbiota in the cytokine responses pattern of splenocytes but not peritoneal macrophages may be associated with physical access of (components of) the intestinal content to splenocytes. In contrast to the peritoneal cavity, the spleen is an integral part of the blood circulation. In addition to accessibility, the immune cell composition and function of the spleen differs from peritoneal macrophages. The spleen contains multiple populations of leukocytes from both the innate and adaptive immune system, whereas peritoneal macrophages belong to the innate immune system. The increased secretion of IL-10 and IL-22 by splenocytes upon BMT suggests an elevated adaptive immune response, as these cytokines are mainly secreted by T cells. However, it remains to be investigated whether the microbiota affect specific subsets of immune cells from the adaptive or innate immune system in the spleen.

The higher response pattern of splenocytes after BMT did not coincide with detectable changes in gut microbial composition as determined by 16S sequencing. It is possible that the resolution of the 16S sequencing technique to distinguish bacterial taxa was not sufficient to show such alterations. Metagenomic sequencing in this respect might have revealed differences at the species level. However, the intra-individual variation in microbial composition between the mice at baseline before BMT-treatment was extremely large. This more than likely hampered detection of significant changes in microbial composition induced by the BMT.

To identify mechanisms underlying the higher response pattern of splenocytes induced by gut microbiota, we further focused on alterations in the function of the gut microbiota induced by BMT. We considered that multiple species of bacteria can exert the same function and produce the same metabolites. For instance, multiple bacterial species can produce SCFA which can cross the intestinal border and have extra-intestinal immune modulatory effects in several tissues including the spleen^8,15^. By performing 1H-NMR on the cecum content samples that were used to inoculate the germfree mice and subsequent unsupervised clustering on the mice, we observed significant heterogeneity in the experimental groups. However, the mice from the control group largely clustered together and are overall different from the other groups. These data indicate that both the BMT and co-housing procedure affected metabolite levels in the gut. Although we can conclude that BMT and co-housing led to alterations in microbial function in our study, the underlying mechanisms linking the gut microbiota to splenocyte cytokine response pattern remain to be elucidated.

In addition to bacterial metabolites, bacterial components such as LPS and peptidoglycans can modulate the immune system. BMT conditioning results in intestinal damage with the likely consequence of LPS leaking into the system. Our data show that the response pattern of splenocytes of BMT-treated mice was transferrable to splenocytes of healthy controls which have no damaged intestine. This makes LPS leakage an unlikely cause for the hyper-responsiveness of the splenocytes in the healthy controls co-housed with BMT mice. On the other hand, bacterial peptidoglycans have been reported to translocate from the gut into the circulation also under basal conditions^6^. It remains to be investigated whether peptidoglycans can explain the hyper-responsiveness of splenocytes after BMT.

Although we conclude that the microbiota are at least partly responsible for the long term transfer of splenocyte cytokine response patterns via co-housing and cecum transplantation, we cannot exclude that other factors are involved. Recently, Liu et al.^15^ identified microRNAs (MIR) in feces that are produced by epithelial cells in the intestine and affect microbial function by regulating bacterial gene transcription. It is thus possible that BMT-induced alterations in epithelial cell MIR secretion play a role in the altered responsiveness of splenocytes after BMT.

Our study may have clinical implications. Here, we focussed on the role of microbiota in syngeneic BMT. However, in humans allogenic BMT is common practice and is associated with graft versus host disease (GVHD). In GVHD, donor immune cells (mainly the T cells) are activated by recipient cells and cause severe inflammation and damage to skin, liver, hematopoietic system and gut. The initiation of GVHD depends not only on activation of the donor T cells, but also on activation of the recipient’s antigen presenting cells and the interaction between these two. As the current study shows, also gut changed microbiota influence the recipient’s immune response and could affect GVHD activation and outcome. Further research is needed to monitor gut microbiota dysbiosis and the possibility of restoring the gut microbiome to a healthy state in BMT patients to reduce the risk of GVHD development.

In conclusion, gut microbiota increase the cytokine responses pattern of splenocytes after BMT. This phenotype can be transferred to splenocytes of healthy controls by co-housing or to germfree mice via transfer of cecum content, indicating that they are independent of BMT-induced intestinal damage and microbial leakage. Whether the BMT-induced higher cytokine response pattern of splenocytes is due to changes in microbial composition or activity, or other transferable factors, remains to be further investigated.

## Methods

### Bone marrow transplantation and co-housing experiment with mice

6 week old male C57Bl/6J mice were purchased from Charles River (Maastricht, The Netherlands) and housed under standard conditions with free access to water and food. After a period of 2 weeks acclimatisation, half of the mice underwent syngeneic BMT. Already before the BMT procedure and during the whole experiment, the BMT-treated mice were co-housed with BMT-treated mice or healthy control mice. This resulted in four different experimental mouse groups: (1) healthy control mice co-housed with healthy control mice, (2) BMT-treated mice co-housed with BMT-treated mice, (3) BMT-treated mice co-housed with healthy controls, (4) healthy controls co-housed with BMT-treated mice. For the BMT procedure, mice received 8 Gy X-ray radiation using an Orthovolt and the day thereafter they received an intravenous injection with donor bone marrow cells in the tail vein. The donor mice were male C57Bl/6J mice of similar age. All mice, both BMT-treated and control mice, received water containing antibiotics (Amphotericin B, Ciprofloxacin, Polymyxin B) from 3 days before until 4 weeks after BMT and were exposed to the same dietary regimen. After 8 weeks recovery on chow diet, mice were fed a low-fat diet (10% energy derived from lard fat; D12450B, Research Diet Services, Wijk bij Duurstede, The Netherlands). Body weight was measured weekly during the entire experiment. Fresh fecal samples from individual mice were obtained 1 week before and 24 weeks after BMT by colon massaging. Each fecal sample was separately stored in a cryovial and snap frozen in liquid nitrogen immediately after collection and subsequently stored at − 80 °C until time of genomic DNA isolation. Mice were euthanized after anesthetization by a subcutaneous injection of a mixture of Neurotranq, Midazolam and Fentanyl. The spleen, peritoneal macrophages and cecum content were collected. This study was part of a larger study of which recently the metabolic characterization was described^14^. All experiments were approved by the animal ethics committee of the Leiden University Medical Center, Netherlands (protocol no. 121031), and conducted in accordance with the European directive 2010/63/UE. This study was carried out in compliance with the ARRIVE guidelines.

### Experiments with germfree mice

Eight to twelve weeks old male germfree Swiss/NIH mice were obtained from Taconic Farms and kept in sterile flexible plastic isolators (Standard Safety Equipment) with free access to sterile water and food. Conventional Swiss/NIH mice were obtained from the local Animal Facility at Universidade Federal de Minas Gerais, Brasil. The cecum content of the mice from the four different experimental groups (1—healthy control mice co-housed with healthy control mice, 2—BMT-treated mice co-housed with BMT-treated mice, 3—healthy controls co-housed with BMT-treated mice, 4—BMT-treated mice co-housed with healthy controls. All mice in the four groups received water containing antibiotics (Amphotericin B, Ciprofloxacin, Polymyxin B) from 3 days before until 4 weeks after BMT and were exposed to the same dietary regimen) were diluted in PBS (10% w/v) and administered by intragastric gavage (200 µL per mice) to the germfree mice. After 2 weeks of microbiota reconstitution, the colonized mice were euthanized to collect spleen and peritoneal macrophages. Spleen and peritoneal macrophages were also obtained from germfree mice and conventional mice of similar age.

### Ex-vivo stimulations of splenocytes

Spleen cells were isolated by gently passing spleens through a sterile 70 μm filter chamber. After washing with sterile PBS and centrifugation at 4 °C (1700 rpm for 10 min), cells were counted using a Z1 Coulter Particle Counter (Beckman Coulter, Woerden, The Netherlands), and subsequently cultured in 24-wells plates (Costar, Corning, the Netherlands) at 5 × 10^6^ cells/well in RPMI-1640 containing 1 mM pyruvate, 2 mM l-glutamine, and 50 mg/L gentamicin, in the presence of 10% fetal calf serum (FCS). Different stimuli were added in a final volume of 1 mL. Splenocytes were stimulated with LPS 10 ng/mL, Polyhydroxyalkanoates (PHA) 10 µg/mL, Polyinosinic:polycytidylic acid (poly(I:C)) 50 µg/mL, heat killed *C. conidia* 1 × 10^6^/mL, heat killed *S. typhimurium* 1 × 10^7^/mL or heat killed *S. aureus* 1 × 10^7^/mL. Supernatants were collected after 48 h for IL-10 and TNF-α determination and after 120 h for IL-22 determination. Supernatants were stored at − 80 °C until concentrations of cytokines were measured.

### Ex-vivo stimulations of peritoneal macrophages

Peritoneal macrophages were isolated from mice by injecting 10 mL of ice-cold sterile PBS (pH 7.4) into the peritoneal cavity, as previously described^16^. After centrifugation and washing, cells were resuspended in RPMI-1640 culture medium containing 1 mM pyruvate, 2 mM l-glutamine, and 50 mg/L gentamicin. Cells were counted and cultured in 96-well round-bottom microtiter plates (Costar, Corning, The Netherlands) at 1 × 10^5^ cells/well, in a final volume of 200 μL. The stimuli were the same as for the splenocytes except that instead of PHA, Pam3Cys was used at 10 µg/mL. Supernatants were collected after 24 h incubation and stored at − 80 °C for measurement of TNF-α and IL-6.

### Cytokine measurements

Cytokine concentrations were measured in supernatants of the ex-vivo stimulation experiments. TNF-α concentrations were determined by a specific radioimmunoassay as previously described^17^. IL-6 was measured using a commercially available ELISA kit (Thermo Fisher Scientific, Waltham, MA, USA). Similarly, IL-22 and IL-10 concentrations were measured using commercially available ELISA kits (R&D Systems, Minneapolis, MN, USA). All according to the instructions of the manufacturer.

### 16S rRNA gene sequencing and data analysis

From the fecal samples, genomic DNA was extracted using phenol:chloroform:isoamylalcohol (25:24:1) (Invitrogen), precipitated with isopropanol, and washed with 70% ethanol. The DNA samples were sent to the Broad Institute of MIT and Harvard (Cambridge, USA) for 16S rRNA gene sequencing. Microbial 16S rRNA gene was amplified targeting the hyper-variable region V4 using forward primer 515F (5′-GTGCCAGCMGCCGCGGTAA-3′) and the reverse primer 806R (5′-GGACTACHVGGGTWTCTAAT-3′). The cycling conditions consisted of an initial denaturation step at 94 °C for 3 min, followed by 25 cycles of denaturation at 94 °C for 45 s, annealing at 50 °C for 60 s, extension at 72 °C for 5 min, and a final extension at 72 °C for 10 min. Sequencing was performed using the Illumina MiSeq platform generating paired-end reads of 175 bp in length in each direction. Overlapping paired-end reads were subsequently aligned. Details of this protocol have been described previously^18^.

Raw sequence data quality was assessed using FastQC, version: 0.11.2 (http://www.bioinformatics.babraham.ac.uk/projects/fastqc/). Reads quality was checked with Sickle, version: 1.33 (https://github.com/najoshi/sickle) and low quality reads were removed. For visualising the taxonomic composition of the fecal microbiota and further beta diversity analysis, QIIME, version: 1.9.1 was used^19^. In brief, closed reference OTU picking with 97% sequence similarity against GreenGenes 13.8 reference database was done. Jackknifed beta-diversity of unweighted UniFrac distances with 10 jackknife replicates was measured at rarefaction depth of 20,000 reads/sample.

### Metabolites measurement by 1H-NMR

Weighed cecum content samples (28.4 ± 8.9 mg) were mixed with 5 volumes of milliQ water and prepared and measured using 1H-NMR spectroscopy as described previously^20^. The identification of metabolites was performed using the databases from Bruker (Bruker Biospin Ltd.) and Chenomx (Chenomx NMR suite 8.2) and assignments were verified by 2D 1H-NMR experiments of selected samples. The quantification of metabolites was performed with the Chenomx software and quantities were corrected for sample weight^21^.

### Statistics

Data are presented as means ± SEM. Experimental groups were compared using Mann–Whitney U test. All statistical analyses were performed using GraphPad Prism version 6 (GraphPad software, San Diego, CA, USA).

## Supplementary Information

Supplementary Figure 1.
