# Supplementary material for: Bone marrow transplantation induces changes in the gut microbiota that chronically increase the cytokine response pattern of splenocytes
Source: Sci Rep. 2022 Apr 27;12:6883. doi: 10.1038/s41598-022-10637-7 (PMC9046407; doi:10.1038/s41598-022-10637-7)
Supplement: Supplementary file 1 — Supplementary Figure 1. [file 41598_2022_10637_MOESM1_ESM.docx]

**Supplementary figure for**

**Bone marrow transplantation induces changes in the gut microbiota that chronically increase the cytokine response pattern of splenocytes**

Saeed Katiraei^1,2^, Janna A. van Diepen^3^, Luciana P. Tavares^4^, Lisa R. Hoving^1,2^, Amanda Pronk^1,2,5^, Ineke Verschueren^3^, Patrick C.N. Rensen^2,5^, Jaap Jan Zwaginga^6^, Sarantos Kostidis^7^, Martin Giera^7^, Mauro Teixera^4^, Ko Willems van Dijk^1,2,5*^, Mihai G. Netea^3^_,_ Jimmy F.P. Berbée^2,5#^, Vanessa van Harmelen^1,2#^

^#^These authors contributed equally to this work.

^1^Department of Human Genetics, ^2^Einthoven Laboratory for Experimental Vascular Medicine, Leiden University Medical Center, Leiden, The Netherlands. ^3^Department of Internal Medicine, Radboud UMC Nijmegen, The Netherlands. ^4^Laboratory of Immunopharmacology, Department of Biochemistry and Immunology, Universidade Federal de Minas Gerais, Belo Horizonte, Brazil. ^5^Department of Medicine, division of Endocrinology, ^6^Department of Immunohematology and Blood Transfusion, ^7^Center for Proteomics and Metabolomics Leiden University Medical Center, Leiden, The Netherlands.

***Corresponding author:**

Ko Willems van Dijk (KwvD)

E-mail: [kowvd@lumc.nl](mailto:kowvd@lumc.nl)


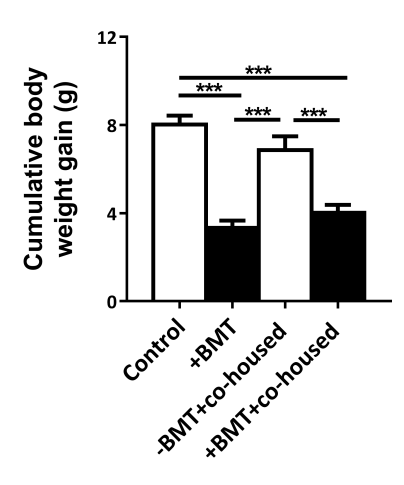

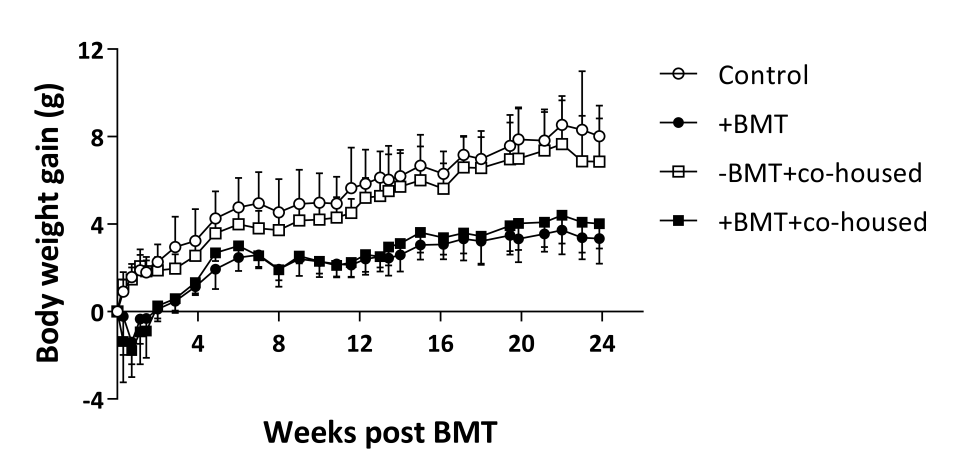
 Supplementary Figure 1. Co-housing BMT treated mice with healthy control mice did not affect body weight. Mice underwent BMT at time point 0 weeks. From 0 weeks until week 8, mice were fed a chow diet. From time point 8 weeks on, mice were fed a 10% LFD. Body weight gain was reduced by the BMT-treatment over the period of 16 weeks LFD, but not affected by co-housing control mice with BMT-treated mice. Values are means ± SEM; Groups were compared using Mann-Whitney U test; n=7-12; ***p<0.001.

**Supplementary Figure 1**

**a**

**b**
